# Ixovex-1, a novel oncolytic E1B-mutated adenovirus

**DOI:** 10.1038/s41417-022-00480-3

**Published:** 2022-05-20

**Authors:** Mohiemen Anwar, Maja-Louise Arendt, Mohanraj Ramachandran, Anette Carlsson, Magnus Essand, Göran Akusjärvi, Ghasan Alusi, Daniel Öberg

**Affiliations:** 1 ENT department, Chelsea and Westminster NHS Foundation Trust, London, UK; 2 Department of Veterinary Clinical Sciences, University of Copenhagen, Copenhagen, Denmark; 3 Department of Immunology, Genetics and Pathology, Rudbeck Laboratory, Uppsala University, Uppsala, Sweden; 4 Department of Medical Biochemistry and Microbiology, Uppsala University, Uppsala Biomedical Centre, Uppsala, Sweden; 5 Ixogen Ltd, London, UK

**Keywords:** Targeted therapies, Gene therapy

## Abstract

There is a great demand for improved oncolytic viruses that selectively replicate within cancer cells while sparing normal cells. Here, we describe a novel oncolytic adenovirus, Ixovex-1, that obtains a cancer-selective replication phenotype by modulating the level of expression of the different, alternatively spliced E1B mRNA isoforms. Ixovex-1 is a recombinant adenovirus that carries a single point mutation in the E1B-93R 3’ splice acceptor site that results in overexpression of the E1B-156R splice isoform. In this paper, we studied the characteristics of this novel oncolytic adenovirus by validating its in vitro behaviour in a panel of normal cells and cancer cells. We additionally studied its anti-tumour efficacy in vivo. Ixovex-1 significantly inhibited tumour growth and prolonged survival of mice in an immune-deficient lung carcinoma tumour implantation model. In complementation experiments, overexpression of E1B-156R was shown to increase the oncolytic index of both Ad5wt and ONYX-015. In contrast to prior viruses of similar type, Ixovex-1 includes a functional E3B region for better in vivo efficacy. Throughout this study, the Ixovex-1 virus has been proven to be superior in competency compared to a virus with multiple deletions.

## Introduction

Oncolytic viruses (OV) selectively replicate in cancer cells that possess specific oncogenic phenotypes, thereby killing cancer cells whilst sparing normal cells. Oncolytic virotherapy is not only an emerging but also an established platform for targeted cancer therapy today [1–3]. Most notably, in October 2015 the American FDA and in December 2015 the European EMA approved a herpes simplex-1 virus (T-Vec, Amgen) as the first oncolytic virus treatment in the western world. A positive and an encouraging step towards establishing OV as an integral platform in the new age of targeted cancer therapeutics.

Attempts have been made to engineer recombinant viruses that replicate selectively in cancer cells. A major target in these experiments is the mammalian p53 tumour suppressor protein, which mediates cell-cycle arrest and/or apoptosis in response to DNA damage or foreign DNA synthesis [4]. Consequently, most viruses, including adenovirus, encode for proteins that counteract those functions in infected cells to allow for efficient viral replication and spread [5]. The two most prominent adenovirus proteins that interact with the p53 pathway are the E1B-55K and E1B-19K, hereafter referred to as 496 R and 176 R, respectively, where R denotes the number of encoded amino acids (aa). 496 R functionally inactivates the p53 protein, whilst 176 R counteracts the pro-apoptotic signalling pathways [6]. Recent studies show that 496 R interacts with more than 90 different cellular proteins [7]. This interaction is achieved via its association with the ubiquitin-proteasome system, RNA metabolism and the E4orf6 adenovirus protein. These factors join together to prevent p53-mediated cell-cycle arrest and programmed cell death in wild-type virus-infected cells. Another important function for 496 R is to counteract the interferon modulated, cell-mediated immune response to viral infections [7]. The E1B locus in general is essential for effective adenovirus DNA replication and subsequent spread.

Oncolytic adenoviruses have commonly been designed to take advantage of the difference in p53 status between neoplastic and normal cells. The E1B-496R protein is a natural target often nulled to remove the binding interactions with p53 by means of deletion within the E1B locus, resulting in an adenovirus that can selectively replicate and ultimately lyse cancer cells that substantially lack p53 activity, but not in cells that possess a normal p53 function. One example is ONYX-015 (originally named dl1520 and also referred to as ONYX-015 or H101), which is a mutant adenovirus that does not express the E1B-496R protein [8] because of a large deletion of the E1B-496R coding sequence and introduction of a stop codon immediately after the translational start site. As a result, this virus lacked the ability to bind and inactivate p53, and thus could supposedly only replicate efficiently in cells defective in p53 function, such as neoplastic cells. Unfortunately, the E1B-496R protein conducts other additional vital functions, besides binding and inactivating p53 [9]. Subsequent studies have shown that the ONYX-015 virus is defective in the transportation of the viral late mRNAs across the host cell nuclear membrane into the cytoplasm, thereby affecting host cell cycle shut-off and translation of late viral mRNAs. Depending on the genetic background of the respective tumour cell line this loss is complemented to some level and therefore ONYX-015 replication is supported, although to a varying degree. Thus, the mutation in ONYX-015 hampered the ability of the mutant virus to reproduce efficiently in many tumour cell lines. An additional problem of ONYX-015 is that the large deletion in E1B-496R has a destabilising effect on the viral genome and packaging of viral progeny [6].

**Fig. 1:**
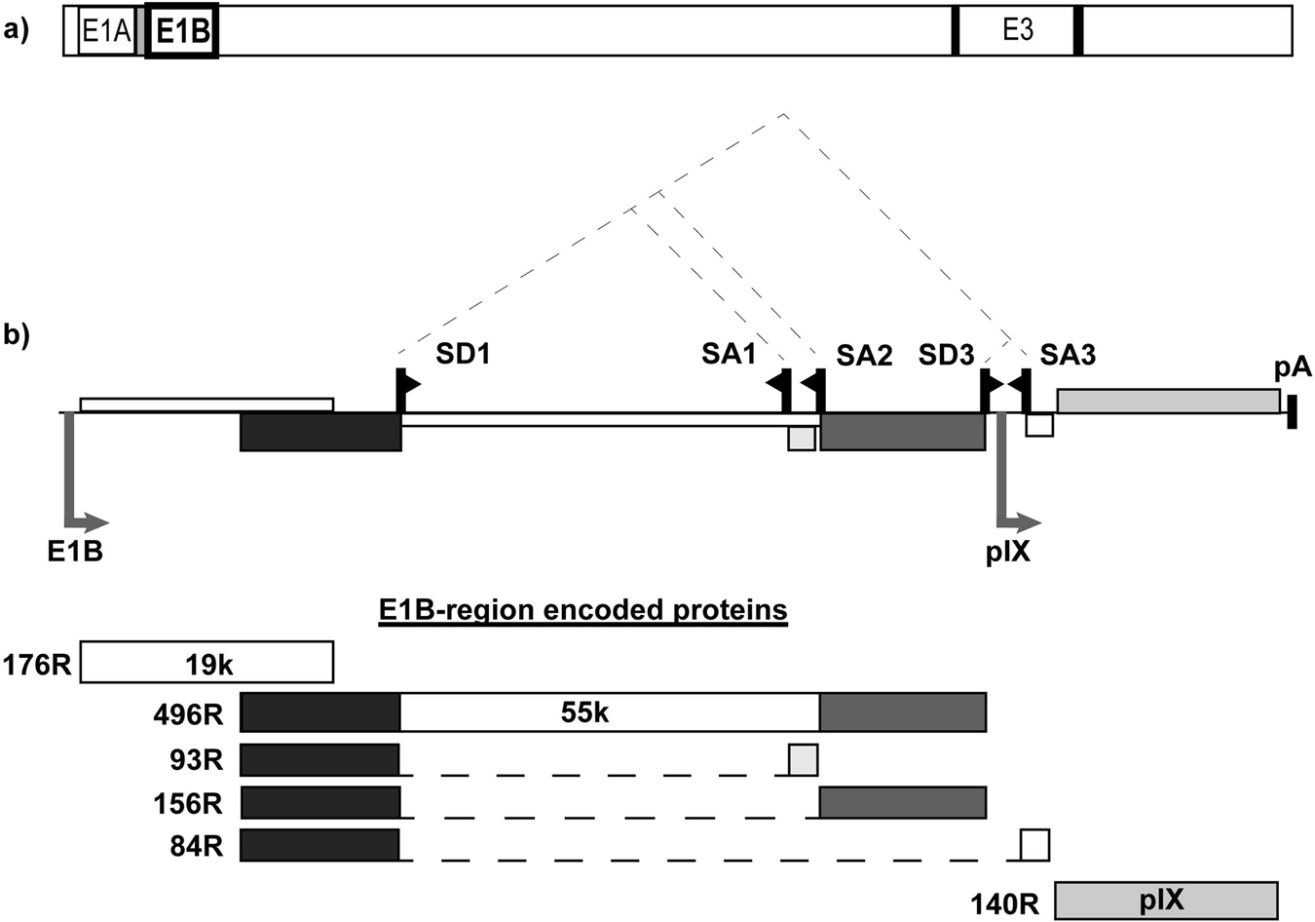
The adenovirus genome and E1B cassette. **a** The full-length genome with the E1 and E3 gene cassettes is indicated. **b** E1B splice map showing alternative E1B gene products. The full-length E1B transcript carries the E1B-176R, E1B-496R and pIX ORF. Through alternative splicing within the E1B-496R ORF, another three additional mRNAs are produced encoding for proteins, E1B-93R, E1B-156R and E1B-84R. The spliced E1B products have the 79 N-terminal aa in common with E1B-496R but differ in the C-terminal, except for E1B-156R, which splices in frame with E1B-496R.

There remains a great demand for newly studied and improved mutant viruses, whose oncolytic ability has been enhanced and which might be useful in the OV-driven cancer therapeutics, without compromising the overall replication efficacy. Here we present a novel patented oncolytic adenovirus, Ixovex-1, which carries a single point mutation in the E1B-93R 3′ splice acceptor site (SA1 and Fig. 1b). This mutation resulted in an enhanced E1B-156R 3’ splice acceptor site (SA2 and Fig. 1b) usage and as a consequence an increased E1B-156R protein expression. A second finding was that E1B-496R protein expression was reduced to undetectable levels in Ixovex-1-infected cells. This created a setting where the replication potential of Ixovex-1 was largely retained in cancer cells but inhibited in normal cells, i.e., an increase in the oncolytic index (OI) of the virus. Further, in complementation experiments expression of the E1B-156R protein increased the OI of both Ad5wt and ONYX-015. Therefore, we postulate that the upregulation of E1B-156R protein expression in Ixovex-1 substitutes for some of the functions normally provided by the E1B-496R protein. In addition, both Ixovex-1 and Ad5wt significantly inhibited tumour growth and prolonged survival of mice in an immune-deficient lung carcinoma tumour model. In contrast to prior art viruses of a similar type, Ixovex-1 include a functional E3B region for better in vivo efficacy [10].

## Materials and methods

### Tissue culture

All cells were cultured at 37 °C and 5% CO_2_ and were tested regularly for mycoplasma contamination. The cell lines used in this study were: A549 (Uppsala University, not authenticated (NA)), H1299 (Barts Cancer Institute London-UK (BCI), NA), H460 (BCI, NA), Hek293 (BCI, NA), HeLa (BCI, NA), JH293 (BCI, NA), Normal Human Bronchial Epithelial cells (NHBE, Lonza), human Small Airway Epithelial Cells (SAEC, Lonza) and Normal Human Lung Fibroblasts (NHLF, Lonza). Cells were grown in Dulbecco’s Modified Eagle Medium (DMEM) supplemented with 10% (V/V) Foetal Calf Serum (FCS) and 1% penicillin/streptomycin, except for NHBE, SAEC and NHLF that were grown as recommended by the vendor (Lonza). Cell lines were regularly controlled for mycoplasma infection.

### Virus and plasmid construction

Plasmid pTG3602 [11] was used as a template to PCR amplify Ad5 nucleotides 1–5055 with Phusion polymerase (ThermoScientific) using oligonucleotides Ad5start (ccacctcgagttaattaacatcatcaataatataccttattttg) and Ad5wt5055as (gtgggtttaaacggatttggtcagggaaaacatg). The PCR product was cloned into pShuttle (Agilent Technologies) using restriction enzymes NotI and PmeI (NEB) and T4 DNA Ligase (NEB), generating plasmid pS-wt. To produce Ixovex-1 virus, SA1 3’ splice acceptor site was mutated in pS-wt using oligonucleotides Mut93Rs (ccttgcatttgggtaatagaagaggagtgttcctaccttaccaatg) and Mut93Ras (cattggtaaggtaggaacactcctcttctattacccaaatgcaagg) with the PCR Mutagenesis XL kit (Stratagene), generating clone pS-Ixo. Five micrograms of pS-Ixo were linearised using PmeI (NEB), purified by phenol/chloroform extraction and ethanol precipitation. Two hundred nanograms of linearised vector was mixed with 100 ng of plasmid pTG3602 and electroporated into BJ5183 cells (Agilent Technologies). Kanamycin resistant clones were screened by size exclusion. Briefly, the pellet of 1 ml bacterial culture was resuspended in 50 μl H_2_O and treated with 50 μl phenol/chloroform. The mixture was spun for 1 min at 13,000 rpm and the water phase was collected and treated for 5 min with a DNA loading dye containing RNaseH before separation on a 0.7% agarose gel. DNA was prepared from the positive clones (Qiagen Maxi Prep kit) and sequenced to ensure that the SA1 mutation had been introduced. To produce Ixovex-1, 2 μg of a positive clone were digested with PacI (to excise the viral genome), phenol/chloroform treated, ethanol precipitated and transfected into Hek293 cells using the Transfectene reagent (Biorad). Five days post transfection cells were harvested, subjected to three rounds of freeze/thawing and applied to a T175 bottle of A549 cells. For experimental purposes, a CsCl purified stock of Ixovex-1 was produced. For this, a CF-10 (ThermoScientific) was seeded with Hek293 cells and infected at 80% confluence with virus lysate. Three days later the CF-10 was harvested. The cell pellet was frozen/thawed three times, lysate cleared by centrifugation and applied to a 1.25/1.4 g/ml CsCl-cushion and spun at 25,000 rpm for 2 h in an ultracentrifuge. The virus band was collected and rerun (40,000 rpm overnight) on a self-generating 1.35 g/ml CsCl gradient. The virus was collected and dialysed overnight at 4 °C in a buffer containing 50 mM Tris-HCl pH 7.8, 150 mM NaCl, 1 mM MgCl_2_ and 10% glycerol in a Slide-A-Lyzer (ThermoScientific) cassette. All virus titres were measured as the 50% tissue culture infective dose (TCID_50_) (pfu/ml) in JH293 cells, as previously described [10], except for the virus used in replication experiments on SAEC and NHLF where titres were determined as fluorescence forming units (ffu) /ml [12]. Viral DNA was purified from a small aliquot of purified virus stock and the number of viral genomes per μl was determined using a spectrophotometer. The ratios between particle count and TCID50 titre of viruses used herein were all less than 20. Similarly, the Ixo-ctrl virus is the Ixovex-1 virus with the SA1 mutation reverted to wild type, in essence, a wild-type virus based on the pTG3602 backbone.

A codon- and Kozak-optimised ORF of Ad5 E1B-156R was ordered from GenScript. The ORF was subcloned EcoRI/XbaI (NEB) into p3xFLAG-cmv-14 (SigmaAldrich) plasmid (here named pCtrl), without the 3′-FLAG fusion, generating p156Ropt.

### Cytotoxicity assay

We used the MTS assay (CellTiter 96 Aqueous Non-Radioactive Cell Proliferation Assay; Promega) to assess the cytotoxicity of Ixovex-1 and the control viruses. Aiming for cells to be confluent on day 6, 1000–4000 cells/well (depending on the rate of growth) were seeded in a 96-well plate in 90 μl DMEM-5% FCS. Viruses (in 10 μl of medium and 0% FCS) were added 18 h after seeding in 1:10 serial dilutions starting at 10,000 viral particles/cell, together with a positive (just cells with no virus) and negative control (no cells just medium). Six days post infection (dpi), survival was determined using the MTS assay, an Opsys MR reader and Revelation Quicklink 4.04 (DynexTechnologies). Dose-response curves were generated and concentrations killing 50% of cells (EC50-values) were determined using untreated cells as reference. All experiments were performed in triplicate and data presented with Standard Error of Mean (SEM).

### Viral replication assay

Cells (10^5^) were seeded in six-well plates in DMEM-10% FCS ~24 h prior to infection. Two and half pfu/cell were used to infect 50% confluent cells in DMEM-2%FCS. Two hours post infection (hpi), the medium was replaced with 10% FCS medium (primary infection). NHBE, SAEC and NHLF were infected with 1 pfu/cell, 2.5 ffu/cell and 1 ffu/cell, respectively, for 2 h in their respective specific media. At indicated hpi, medium and cells were harvested (by scraping), washed in PBS, freeze/thawed three times using liquid nitrogen and a 37 ° water bath and stored at −80 °C. TCID50 was further determined using a limited dilution assay [10]. For SAEC and NHLF a limited dilution assay based on fluorescence was used [12]. All experiments were performed in triplicate and data presented with SEM.

Oncolytic index (OI) in the complementation experiments was determined based on the replication rates in cancer- compared to normal cells, all transfected (Transfectene) with an expression plasmid (2 µg) with (p156R) or without (pControl) the E1B-156R ORF, as follows: OI = ((a/b)/(c/d)) where a = TCID50 from virus-infected cancer cells transfected with E1B-156R expression plasmid (p156R), b = TCID50 from virus-infected normal (HBEC) cells transfected with E1B-156R expression plasmid (p156R), c = TCID50 from virus-infected cancer cells transfected with 'empty' expression plasmid (pControl) and d = TCID50 from virus-infected normal (HBEC) cells transfected with 'empty' expression plasmid (pControl).

### Western blot

A549 cells were infected with 2.5 pfu/cell and total protein extracted at 48 hpi using RIPA buffer (50 mM TRIS, 150 mM NaCl, 1 mM MgCL_2_, 1% NP40 and 0.1% SDS) complemented with protease inhibitors. Control and mock lanes are lysates from non-infected cells. Protein concentration was determined using the Bradford reagent. Twenty μg total protein from each sample were loaded onto a 10% PAGE gel. The proteins were transferred to a PVDF (BioRad) membrane by wet blotting. The membrane was blocked using 3% BSA-1xTBS solution for 1 h. Primary antibodies used were: Ad capsid proteins (polyclonal Abcam-6982), E1B-496R (2A6, dilution 1:500, [13]), actin (Santa Cruz, I-19) and p53 (Cell Signalling, #9282). All antibodies were diluted as recommended in 1.5% BSA-1xTBS. Membranes were incubated with the primary antibodies for 15–24 h at 4 °C and washed with 1xTBS 3% Tween-20 three times for 10 min. HRP-coupled secondary antibodies against respective primary antibodies were diluted 1:5000 in 1.5% BSA-1xTBS and incubated with the membrane for 1 h. Membranes were washed as above, exposed for 1 min with the ECL Plus (GE, RPN2132) reagent incubated with Hyperfilm (GE) and developed.

### RT-PCR

A549 cells were infected with 2.5 pfu/cell of the respective virus, total RNA was extracted at 48 hpi using Trizol (Invitrogen). The RNA was treated with DNase I (NEB), phenol/chloroform extracted and ethanol precipitated. One μg total RNA and oligo-dT oligonucleotide were used to synthesise cDNA (Invitrogen, SuperScript^®^ III). The cDNA was used as a template in PCR (NEB Taq DNA Polymerase) reactions with a common sense oligonucleotide (496RSense, gcctgctactgttgtcttccg) and either of the following antisense oligonucleotides: 93Ras-cacccccctcctgtacaac, 156Ras-gacatgctctcgggctgtacaac or 84Ras-caaacgagttggtgctcatg. The amplicon length of each is around 200 nucleotides. The PCR reaction was terminated after 20 cycles and an aliquot run on a 2% agarose gel.

### In vivo assay

Nude NMRI mice (6-week-old females, Taconic Denmark) were injected subcutaneously on their right flank with 4 × 10^6^ H1299 cells in 200 μl PBS mixed 1:1 with matrigel (BD biosciences). After 2 weeks, 28 mice with tumour growth (size ~70–100 mm^3^) were randomised into four groups with seven mice in each. Tumours were injected three times at 24 h intervals with 50 μl PBS, or 50 µl PBS containing10^8^ viral particles of Ad5wt, ONYX-015 or Ixovex-1. All tumours were then measured using a digital caliper two times a week. The groups were injected non-blinded. Tumour size was calculated as length × width × depth × 0.66. Data plotted are the volume means ± SEM. One-way ANOVA analysis of variance was performed comparing the tumour volumes at the last common time point (day 23). Survival analysis was performed using Kaplan–Meier survival analysis (*p < 0.05, log-rank test for statistical significance). Each virus group was compared separately to the PBS group (control). *P* values <0.05 were considered significant. The Prism 6 software (GraphPad Software, Inc) was used throughout the analysis. All mice were killed by dislocation of the neck, in a separate room where animals were kept, at the end of the experiment or when tumour burden had reached the endpoint (tumour size >1000 mm^3^). Other criteria were considered to determine the endpoint, but none else than tumour size was found, except in the ONYX-015 group where one mouse had ulceration of the skin covering the growing tumour on day 29. The well-being of mice was checked daily. The mice were kept three or four animals per cage, had a wood chip for burrowing, tissue to shred and carton houses to sleep in. All in vivo work was performed in accordance with the Swedish animal ethical permit C215/12, The FELASA guidelines and pre-cleared by the departmental animal ethics committee. All possible means were taken to minimise the suffering of the animals used in the research reported in this paper.

## Results

### Construction of an Ad5 virus defective in E1B-496R protein expression

The Ad5 E1B region (Fig. 1a) generates a group of alternatively spliced mRNAs that encode for at least five proteins (Fig. 1b). The major unspliced E1B mRNA encodes for the E1B-19K (E1B-176R) protein and from an overlapping reading frame also the E1B-496R protein. Three additional E1B proteins are encoded from mRNAs that are spliced using a 5′ splice site within the E1B-496R reading frame (SD1) and three alternative 3′ splice acceptor sites (SA1 3). Therefore, the E1B-93R, E1B-156R and E1B-84R proteins share a 79 aa N-terminal sequence with the E1B-496R protein. The E1B-93R and E1B-84R proteins have unique C-termini, whereas the mRNA encoding for the E1B-156R protein is spliced in frame with the E1B-496R protein. In our work to probe the function of the E1B proteins we generated a virus mutant, the Ixovex-1 virus, in which the SA1 3′ splice acceptor site sequence CAG:GA was mutated to CGG:GA. This single nucleotide point mutation (genomic location 3216, NCBI ref. AC_000008.1) changed aa 399 in the E1B-496R protein from an arginine codon (AGG) to a glycine codon (GGG). As a control, the SA1 3’ splice acceptor site mutation in Ixovex-1 was reverted to wild type, generating the Ixo-ctrl virus.

### Dynamics in E1B protein and mRNA expression

**Fig. 2:**
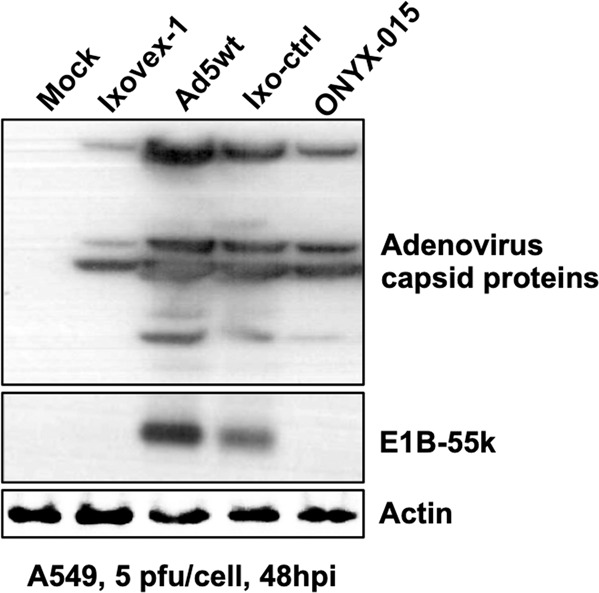
An amino acid change in the E1B-496R protein results in a loss of protein accumulation. A549 cells were infected with the respective virus at 2.5 pfu/cell and total cell lysate was collected at 48 hpi. Shown is a western blot stained with polyclonal α-capsid (late) protein antibody (top panel), a monoclonal α-E1B-496R antibody (2A6, middle panel) and a monoclonal α-actin antibody as a loading control (lowest panel). The Control lane is lysate from non-infected cells.

To study the expression profile of viral proteins, total protein lysates were prepared from Ad5wt, ONYX-015, Ixovex-1 and Ixo-ctrl infected A549 cells at 48 hpi. As shown in Fig. 2, all viruses expressed late protein, i.e., had reached the late phase of adenoviral replication. Surprisingly, the single point mutation in Ixovex-1 resulted in the elimination of E1B-496R protein accumulation (Fig. 2). Further, it is noteworthy that both the Ixovex-1 and ONYX-015 viruses expressed less late proteins compared to the Ad5wt and Ixo-ctrl virus-infected cells. The reduced expression of late proteins in Ixovex-1 and ONYX-015 was not entirely unexpected since the E1B-496R protein serves multiple important functions during a lytic virus infection (reviewed in ref. [6]).

**Fig. 3:**
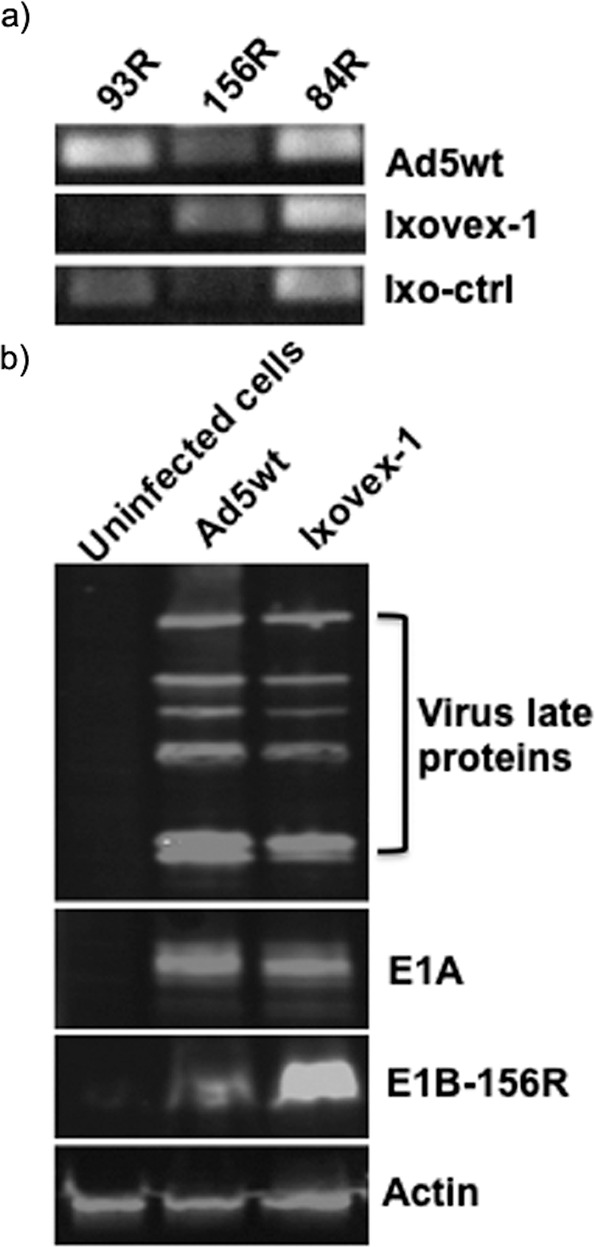
The point mutation in the E1B-496R ORF in Ixovex-1 blocks splicing to the E1B-93R splice acceptor (SA1). A549 cells were infected with the respective virus at 2.5 pfu/cell and **a** total RNA was collected at 48 hpi. cDNA was made using an oligo-dT oligonucleotide. PCR was performed using a common sense oligonucleotide upstream of the splice donor 1 and a specific antisense oligonucleotide downstream of the respective splice acceptor. The PCR reactions were run on a 2% agarose gel. **b** In parallel, total protein was harvested at 48 hpi and western blot was performed using the 2A6 antibody that binds the common N-terminus shared by E1B-496R and its smaller spliced versions, an anti-E1A and a polyclonal anti-capsid (late) protein antibody.

As expected, the mutation in Ixovex-1 modifying SA1 also effectively inhibited SA1 site usage (Fig. 3a). Interestingly, the inhibition of the SA1 site resulted in a slight increase in the SA2 site usage (Fig. 3a) and a substantial increase in E1B-156R protein accumulation (Fig. 3b). In the revertant (Ixo-ctrl), the balance of the SA1/SA2 splicing was restored (Fig. 3a). ONYX-015 could not be used in this experiment since the deletion in this virus removes both the SA1 and SA2 sites (Fig. 1b).

### Ixovex-1 does not induce p53 degradation

**Fig. 4:**
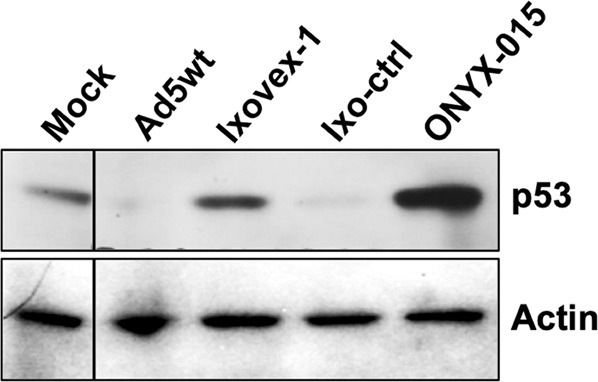
Ixovex-1 fails to induce p53 degradation, but does not induce p53 accumulation. A549 cells were infected with the respective virus at 2.5 pfu/cell and total cell lysate was collected 48 hpi. Shown is a western blot using a monoclonal α-p53 antibody (top panel) and the monoclonal α-actin antibody as a loading control (lower panel). Mock lane is lysate from non-infected cells.

The absence of E1B-496R protein accumulation in Ixovex-1-infected cells (Fig. 2) would be expected to result in a deficiency to degrade p53. To test this prediction A549 cells were infected with the collection of viruses, and p53 protein accumulation was measured at 48 hpi by western blot analysis. As shown in Fig. 4, in Ixovex-1 infected cells, p53 was not degraded compared to Ad5wt-infected cells. Reverting the mutation in Ixovex-1 (i.e., Ixo-ctrl), restored the capacity of the virus to degrade p53. Interestingly, we have consistently observed that the p53 level is higher in ONYX-015 compared to Ixovex-1 infected cells (Fig. 4). Taken together these results suggest that Ixovex-1, because of its lack of E1B-496R protein accumulation (Fig. 2), is defective in p53 degradation but does not induce p53 accumulation (Fig. 4).

### Ixovex-1 replicates efficiently in cancer cells

**Fig. 5:**
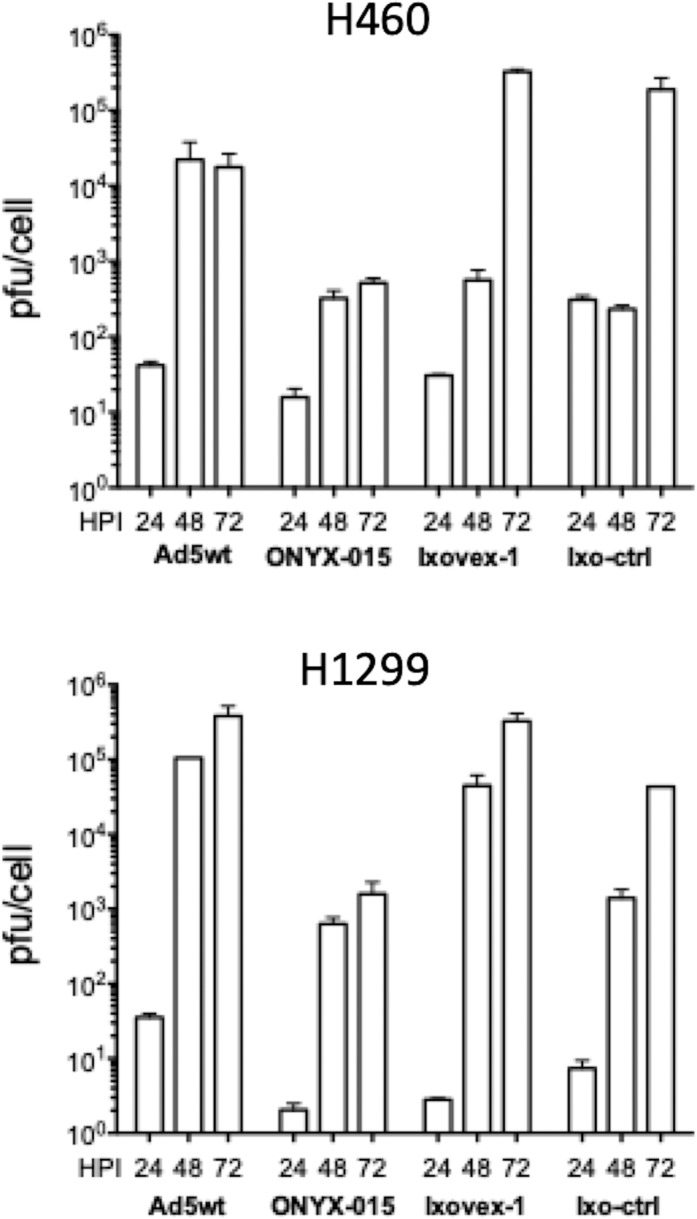
Replication-efficiencies in H460 and H1299 cancer cells. Each cell line was infected with 2.5 pfu/cell of the respective virus. Cells and media were harvested at 24, 48 and 72 hpi and analysed by a limited dilution assay. CPE was scored after 10 days and TCID_50_ (pfu/cell) results were calculated.

Although late protein accumulation appeared to be close to normal in Ixovex-1-infected cells (Figs. 2, 3b), we tested whether the SA1 mutation in Ixovex-1 may perturb an important function required for new virus progeny formation. For this experiment H460 and H1299 cells were infected with Ad5wt, ONYX-015, Ixovex-1 and Ixo-ctrl viruses and new virus formation was titrated at 24, 48 and 72 hpi. As shown in Fig. 5, the Ixovex-1 virus yielded similar burst sizes as Ad5wt and Ixo-ctrl viruses in both cell types. Interestingly, the replication efficiency of Ixovex-1 was 100 to 1000-fold better compared to the ONYX-015 virus.

### Ixovex-1 fails to replicate efficiently in normal cells

**Fig. 6:**
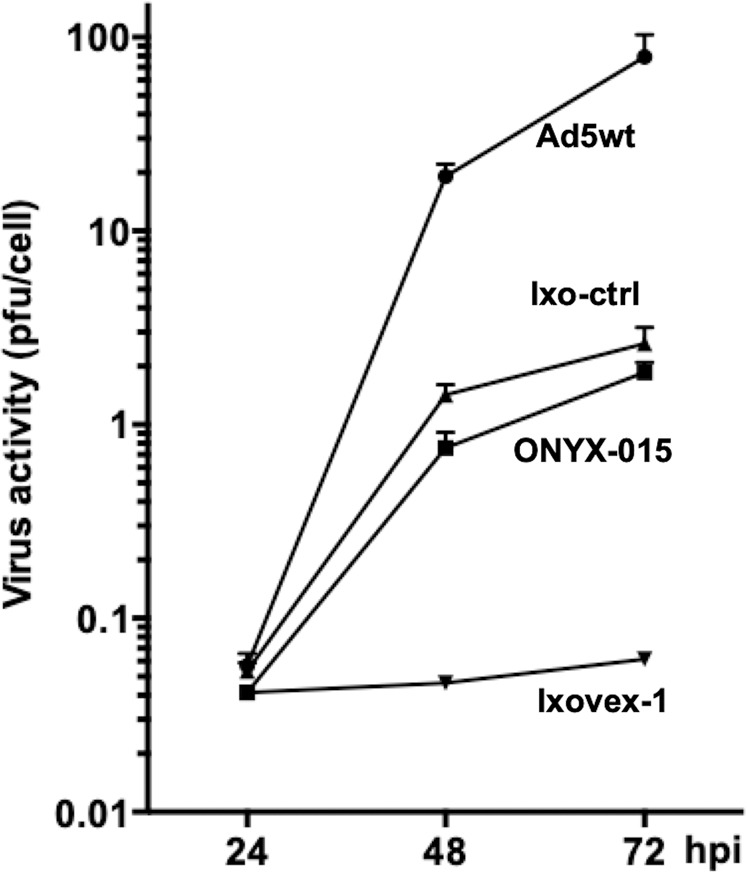
Replication-competencies in NHBE. Cells were infected with 1 pfu/cell of the respective virus. Cells and media were harvested at 24, 48 and 72 hpi and analysed by a limited dilution assay. CPE was scored after 10 days and TCID_50_ (pfu/cell) results were calculated. The result for Ixovex-1 was adjusted upwards because the level of replication was slightly below the detection limit.

To examine the replication capacity of Ixovex-1 in normal cells, Ad5wt, ONYX-015, Ixovex-1 and Ixo-ctrl viruses were used to infect NHBE. As shown in Fig. 6, Ixovex-1 generated much less virus progeny at all time points tested, whereas Ixo-ctrl and ONYX-015 showed an intermediate replication capacity in NHBE compared to Ad5wt. In fact, these differences in replication capacity might even be more pronounced since Ixovex-1 replication barely reached the detection limit in the TCID50 experiment at all time points, with no sign of replication at 24 h, barely detectable at 48 h and some increase at 72 h. The replication capacity of Ad5wt, ONYX-015 and Ixovex-1 was also examined in SAEC and NHLF (see supplemental material Figs. 1, 2). At 48 and 72 h, there was a 10–15x less replication capacity for both ONYX-015 and Ixovex-1 (with a tendency for even less for Ixovex-1), as compared to Ad5wt.

To further evaluate whether the upregulation of E1B-156R expression mediated an increased oncolytic index (OI), per se, both Ad5wt and ONYX-015 infections were complemented in HeLa, H460 and NHBE by cotransfection with an expression plasmid with or without the Ad5 E1B-156R ORF. Interestingly, the complementation of Ad5wt with E1B-156R increased the OI 3.7 and 44 times in HeLa and H460 compared to NHBE, respectively (Table 1). Additionally, the OI of ONYX-015 was increased 5.5 times in HeLa compared to NHBE.

**Table 1:** Complementing Ad5wt and ONYX-015 with excess E1B-156R protein increases the Oncolytic Index.

	Pfu/cell				
	a (+156 R)	b (+156 R)	c (ctrl)	d (ctrl)	OI = (a/b)/(c/d)
Ad5wt (HeLa vs. NHBE)	3710	5.13	5989	31.0	3.7
Ad5wt (H460 vs. NHBE)	32662	5.13	4447	31.0	44
ONYX-015 (HeLa vs. NHBE)	805	242	1715	2835	5.5

Presented are the raw TCID50 values at 48 hpi and a calculation of the relative decrease in efficacy in normal cells versus cancer cells termed Oncolytic Index (OI).

a = TCID50 for cancer cells complemented with 156 R. b = TCID50 for normal (NHBE) cells complemented with 156 R. c = TCID50 for cancer cells with control. d = TCID50 for normal cells with controls.

In agreement with the low replication capacity of Ixovex-1 in NHBE (Fig. 6) the cytotoxicity was dramatically reduced for Ixovex-1 compared to the Ad5wt, ONYX-015 and Ixo-ctrl viruses (Table 2). Thus, Ad5wt was >500-fold, Ixo-ctrl >700-fold and ONYX-015 >35-fold more toxic to normal cells compared to Ixovex-1.

**Table 2:** Ixovex-1 is more than 500-fold less cytotoxic to normal cells compared with the unmodified virus (Ad5wt).

	Ad5wt	ONYX-015	Ixo-ctrl	Ixovex-1
pfu/cell	0.042	0.63	0.031	23
Relative	1	14.8	0.73	535

Presented are the raw EC50-values (top row) and the fold inhibition of cytotoxicity in relation to Ad5wt (bottom row).

### Ixovex-1 reduces tumour growth in nude mice

**Fig. 7:**
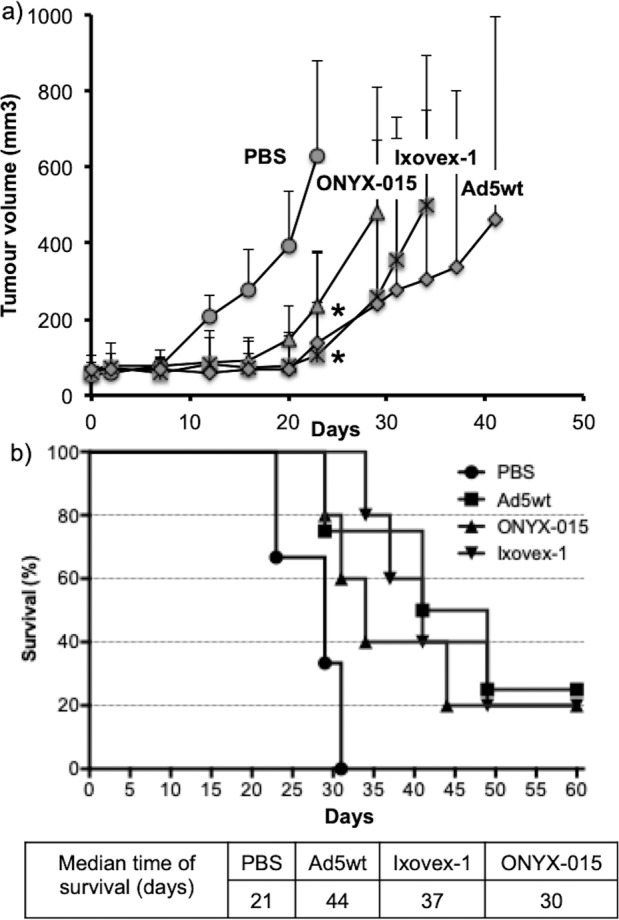
Ixovex-1 significantly inhibits tumour growth in nude mice and prolongs survival. Nude NMRI mice were injected subcutaneously with H1299 cells in PBS mixed 1:1 with matrigel. After 2 weeks, mice with tumour growth (size ~70–100 mm^3^) were randomised into four groups with seven mice in each. Tumours were injected on days -2, -1, and 0 with (10^8^ viral particles) Ad5wt, ONYX-015, Ixovex-1 or PBS only. **a** All tumours were measured using a digital caliper two times per week. Tumour size was calculated as: length × width × depth × 0.66. Mice were sacrificed when tumour size exceeded 1000 mm^3^. Data plotted are the volume means ± SEM. One-way ANOVA analysis of variance was performed comparing the growth curves. Prism 6 software (GraphPad Software Inc.) was used throughout the analysis. **b** The proportions of live mice are plotted against time. The median time of survival was determined by Kaplan–Meier survival analysis (*p < 0.05, log-rank test for statistical significance).

To determine whether Ixovex-1 inhibited tumour progression in vivo, tumours were grown from H1299 cells injected on the flanks of 6 weeks old NMRI nude mice. After 2 weeks, tumours (70–100 mm^3^) were injected at days -2, -1 and 0 with a single dose (10^8^ viral particles) per time point of Ixovex-1, Ad5wt, ONYX-015 or PBS only. As shown in Fig. 7a, Ixovex-1 and Ad5wt were better in inhibiting tumour growth compared to ONYX-015, and significantly better compared to the PBS control group, *p* < 0.05 (One-way ANOVA analysis on tumour volumes only at day 23). Two mice in each virus injected group showed complete remission. After 3 weeks with no sign of tumour regrowth, they were considered disease-free. Two mice of the Ad5wt group had stable disease, i.e. small tumours with a size that did not increase for 3 consecutive weeks, but tumour growth was reinitiated and tumour sizes had increased drastically during the last 2 weeks of the study. The median time of survival within each virus injected group (44 days in Ad5wt, 37 days in Ixovex-1 and 30 days in ONYX-015) was significantly increased compared to the PBS control group (21 days, *p < 0.05), as determined by Kaplan–Meier survival analysis (Fig. 7b).

## Discussion

During our work to construct novel oncolytic adenoviruses we introduced a large set of different mutations into the E1B region of the Ad5 genome. In this work, we constructed a viral clone with a single point mutation at position 3216. This mutation inactivated the E1B-93R 3′ splice acceptor site (SA1 and Fig. 1b). In addition, this point mutation also changed E1B-496R aa codon 399 from an arginine to a glycine (R399G). This virus was named Ixovex-1. Here we characterise E1B gene expression and validate the oncolytic potential of this virus. Although there are other adenoviral mutants available with changes in the E1B region, the Ixovex-1 virus differs by encoding for a wild-type E3B region. This region is essential in the initial phase of the viral infection to avoid early clearance of the virus by the immune system [10].

Our results show that the Ixovex-1 mutation leads to a lack of E1B-496R protein accumulation in infected cells (Fig. 2). We believe that this is because the R399G mutation destabilises the E1B-496R protein. Others have introduced aa changes into E1B-496R and several of these were shown to destabilise the protein [14]. Accumulation of p53 was seen in both Ixovex-1- and ONYX-015 infected cells, but not in Ad5wt-infected cells (Fig. 4).

We showed enhanced splicing to the E1B-156R 3′ splice acceptor site (SA2 and Fig. 3a) results in an increase in E1B-156R protein expression in Ixovex-1-infected cells (Fig. 3b). Splicing between SD1 and SA2, generating the E1B-156R mRNA, not only removes a large part of the E1B-496R ORF but is also in-frame. Thus, the splicing event removes 340 internal aa from the E1B-496R protein leaving the C-terminal 77 aa fused to the 79 N-terminal aa. The Dobner lab has previously shown that the E1B-156R protein retains some ability to inhibit p53 through its C-terminus [15]. It is also possible that E1B-156R retains other functions associated with the E1B-496R protein. The E1B-496R and E1B-156R proteins interact with many of the same cellular proteins [15–18]. E1B-496R has been assigned several functions besides participating in p53 degradation. It is also connected to the regulation of the selective nuclear export of late viral mRNA [19, 20], inhibition of cellular mRNA translation and promotion of late viral mRNA translation [21]. The E1B-496R forms a physical complex with the viral E4orf6 protein [22]. This complex is important for many of the functions associated with E1B-496R. The E1B-156R protein has also been shown to interact with the E4orf6 protein. In addition, similar to E1B-496R it also interacts with Daxx [15]. The E1B-156R might compensate for some of these E1B-496R functions, which fit with the increased expression of E1B-156R by Ixovex-1 and its enhanced profile in cancer cells when compared to the ONYX-015 virus. Sieber et al. [15] speculated that functions of E1B-156R in addition to its interaction with Daxx and E4orf6 might also enable it to affect functions of E1B-496R around these proteins. Interaction with cellular and viral factors by E1B-156R, shared with E1B-496R, could lead to competition. Interestingly, Ad2 E1B-496R, which is almost identical to the Ad5 counterpart, forms dimers [23]. This enables further possibilities, such as modification of E1B-496R through heterodimer formation, affecting the multitude of E1B-496R functions and interactions.

In normal cells, the toxicity of each virus largely mirrored virus replication capacity. The decrease in toxicity and the inhibition of replication in normal cells indicate an astounding safety profile of Ixovex-1. The observation that the ONYX-015 virus replicated better in normal cells compared to Ixovex-1, clearly in NHBE, early infection in SAEC (24 h), and to the same degree in NHLF (Supplemental Figs. 1, 2), is intriguing considering that the ONYX-015 virus carries a large deletion that removes the coding sequences and splice signals needed to express the E1B-496R, E1B-93R and E1B-156R proteins [24]. Interestingly, the difference in replication capacity in normal cells (Fig. 6) between ONYX-015, Ixo-ctrl and Ixovex-1 was not seen in cancer cells (Fig. 5). This result indicates that Ixovex-1 infection in normal cells has become severely restricted. There might be a block already at the early stage of infection giving the cells time to better fight the virus. Miller et al. showed that when p53 accumulates in E1B-496R-null virus-infected cells it is not the transcription activating effect of p53 that mediated the inhibition to virus replication. Instead, they found the upregulation of 340 genes enriched for those associated with immune responses and anti-viral defence [25]. More specifically, the set of genes contained many interferon-stimulated genes (ISGs). It has also been demonstrated that E1B-496R represses the expression of ISGs and blocks type I interferon-induced inhibition of viral DNA synthesis and replication in normal human cells [26].

In contrast, cancer cells might compensate for the reduced E1B-496R function(s) in combination with complementation by the overlapping functions of E1B-496R and E1B-156R. These results indicate that it could be the change in the balance of E1B gene expression that has an impact on the attenuation of Ixovex-1 in normal cells in comparison to the other viruses. This hypothesis is strengthened by the observation that the supplementation of E1B-156R to both Ad5wt and ONYX-015 infection increases the OI of the respective viruses (Table 1).

An advantage of the Ixovex-1 virus in comparison to other patented adenovirus of similar type is that the ONYX-015 [8], -051 and -053 [27] all are missing the E3B gene region. This region was originally deleted to enhance the safety profile of ONYX-015. It was later found that elimination of this region resulted in premature clearance of the virus by the host immune system [10].

The adenovirus family is divided into seven species, named A-G, with a total of more than 100 different sero/genotypes described. We believe that the splicing pattern seen in Ad5, which belongs to species C, is conserved among all adenovirus types described and that the advantageous oncoselectivity described here for Ad5 caused by mutations of a splice site would be mirrored in most if not all of the other serotypes.

The overall higher efficacy of the Ad5wt virus in the assays is likely a result of Ixovex-1 and Ixo-ctrl being based on the wild-type strain pTG3602 [28]. This backbone carries a few point mutations scattered throughout the genome [11]. However, none of the mutations are within the first 5000 base pairs of the Ad and hence not affecting the E1B region. The effect of these mutations has not been examined.

We have shown that Ixovex-1 significantly inhibits tumour growth in a human lung carcinoma animal model and that ONYX-015 does not (Fig. 7a). This mirrors Ixovex-1’s relative higher replicative capacity in the same cells (H1299) in vitro (Fig. 5). Although tumour growth was significantly inhibited by Ixovex-1, as opposed to ONYX-015, two mice out of seven turned out disease-free in both groups. The H1299 cells are fairly aggressive as an animal tumour model. Few cells surviving the oncolytic effect will enable the tumour to regrow quickly. We have used a relatively low dose of virus in this initial in vivo experiment, 10^8^ virus particles. In other studies, significantly higher doses were needed to attain the desired effect. Thus, doses of up to 5 × 10^10^ viral particles/injection have been used. This finding is encouraging for further preclinical work and potential future clinical trials using Ixovex-1.

A multitude of adenovirus variants have been described with enhanced cancer-killing properties [29]. It should be noted that the Ixovex-1 mutation can theoretically be combined with all of these described mutations, except for a few E1B-deleted virus. This makes Ixovex-1 a good platform for the future creation of multi-enhanced adenovirus systems and targeted cancer therapeutics.

## Supplementary information

Supplemental material

## Data Availability

All data generated or analysed during this study are included in this published article [and its supplementary information files].
