# Supplementary material for: Ixovex-1, a novel oncolytic E1B-mutated adenovirus
Source: Cancer Gene Ther. 2022 May 20;29(11):1628–35. doi: 10.1038/s41417-022-00480-3 (PMC9663300; doi:10.1038/s41417-022-00480-3)
Supplement: Supplementary file 1 — Supplemental material [file 41417_2022_480_MOESM1_ESM.pdf]

sFig. 1

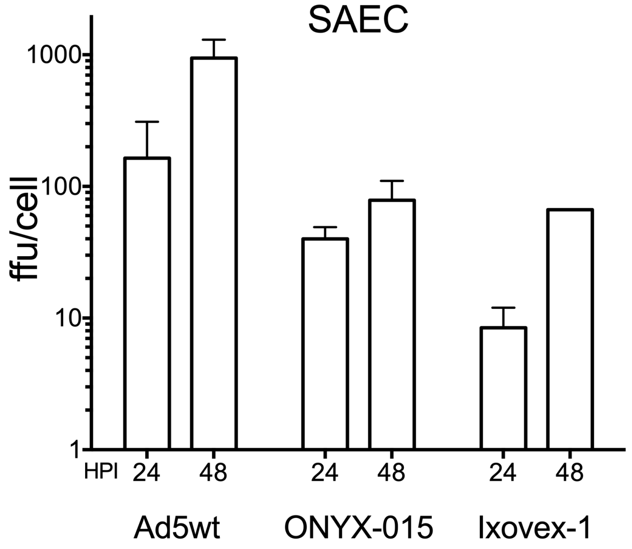

Supplemental figure 1. Replication-competencies in SAEC. Cells were infected with 2.5 ffu/cell of respective virus. Cells and media were harvested at 24 and 48 hpi and virus activity determined by a fluorescence based limited dilution assay (ffu/cell).

sFig. 2

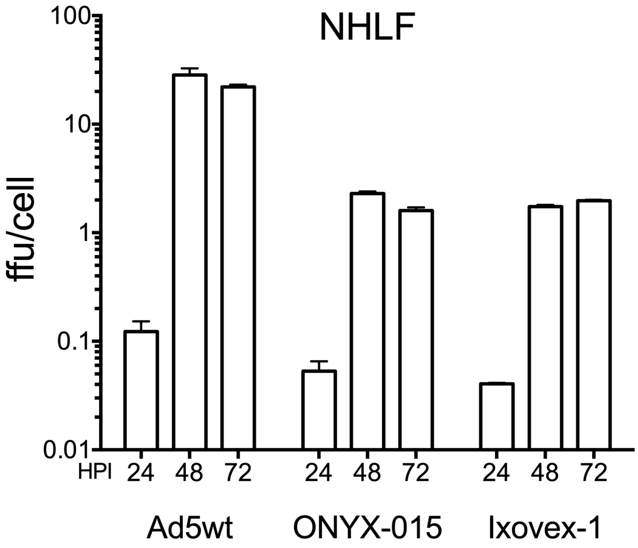

Supplemental figure 2. Replication-competencies in NHLF. Cells were infected with 1 pfu/cell of respective virus. Cells and media were harvested at 24, 48 and 72 hpi and virus activity determined by a fluorescence based limited dilution assay (ffu/cell).
